# Otorhinolaryngological manifestations associated to gastroesophageal reflux. An ever present theme!!!

**DOI:** 10.1016/S1808-8694(15)31058-2

**Published:** 2015-10-22

**Authors:** Regina Helena Garcia Martins

**Affiliations:** Associate Professor of Otorhinolaryngology of the Botucatu Medical School – Unesp

Gastroesophageal Reflux Disease (GERD) is defined as a “chronic disease caused by the backflow of part of the gastroduodenal content to the esophagus or adjacent organs, causing a variable range of esophageal and/or extra-esophageal signs and symptoms either associated or not with tissue lesions”.

This topic started to be approached in 1946 when Allison introduced the term reflux esophagitis. Two decades later, Cherry and Margulies, in 1968, described laryngopharyngeal reflux and contact ulcers for the first time. In the 80's there was a major progress in research, in an attempt to develop more accurate diagnostic techniques, a time when esophageal pH monitoring was deemed the gold standard for reflux diagnosis. Parallel to the search of the best diagnostic method, the pharmaceutical industry worked hard to develop increasingly more efficient drugs to treat the disease, reaching revolutionary formulas such as proton pump inhibitors, which have proven to be efficacious in reducing acid secretion under low toxicity.

The theme is so broad that gastroesophageal reflux otorhinolaryngological manifestations have fostered numerous clinical and experimental research projects. Some studies point towards a reflux prevalence of 4 to 10% in the patients that come to see the otorhinolaryngologist. The classical reflux symptoms, namely heartburn and regurgitation, may be absent in up to 50% of the patients with extra-esophageal reflux. All the airway may be compromised, showing diverse symptoms such as excessive salivation, otalgia, dental enamel erosion, carious lesions, asthma, bronchitis, rhinosinusitis, chronic cough, a feeling of something in the throat, sore throat, hoarseness, dysphagia, laryngospasm, post-nasal dripping, halitosis, vocal fatigue, larynx cancer, subglottic stenosis, stridor, snoring, sleep apnea, non-cardiac related chest pain and sudden death. Of all the symptoms of gastroesophageal reflux, hoarseness is the most frequent, being present in approximately 90% of patients with acidic laryngitis.

Experimental studies have shown lesions in the laryngeal mucosa when exposed to minimum doses of the gastric acid content, even for a short amount of time.

Among the many signs found in the otorhinolaryngological exam, we have: lingual tonsil hyperplasia, rough and dark tongue, posterior glottic commisure erythema, irregularities in vocal fold mucosa, erythema and/or edema of tracheobronchial segments, granulomas, pseudo-groove, contact ulcer, laryngeal mucosa hypertrophy, vocal nodules, vocal polyps, subglottic stenosis, leukoplakia and carcinoma.

Even after 60 years of investigation, both the diagnosis and treatment of GERD and that of the extra-esophageal reflux have been the subject of numerous research projects because of the controversy they bear. Thus, the very gold standard given to pH monitoring in the diagnosis has been questioned by some authors, saying that besides not presenting 100% sensitivity, lead installation in the digestive tract impairs the patients' eating habits, thus harming the results and, consequently, also the diagnosis. For some authors, the diagnostic test with the proton pump inhibitors have proven more efficacious and is based on assessing the treatment response with the use of high drug doses for a short time interval.

In investigating the GERD treatment efficacy and its complications, current studies have compared clinical and endoscopic results obtained with the different proton pump inhibitors, assessing the best dose and the benefits of its association with H2 receptor antagonists. The controversial results of these studies call for additional research – which makes the topic ever present.

